# Safety and effectiveness of diet and detox teas for weight loss: a mini-review

**DOI:** 10.3389/fnut.2026.1777795

**Published:** 2026-04-09

**Authors:** Fouzia Noor, Reyusha Chalise, Alvin Tran

**Affiliations:** Department of Population Health and Leadership, School of Health Sciences, University of New Haven, West Haven, CT, United States

**Keywords:** adverse health effects, consumer safety, detox teas, diet teas, nutrition misinformation, slimming tea, weight management

## Abstract

Diet and detox teas are commonly marketed as over-the-counter products that claim to support weight loss through detoxification and metabolic enhancement. Despite their widespread use, the evidence base supporting these claims remains unclear. This mini review summarizes and critically examines the existing literature on the safety and efficacy of diet and detox teas marketed for weight loss. A focused review of the literature identified 10 studies spanning experimental research, case reports, product analyses, and marketing assessments. Overall, the available evidence provides limited support for weight loss benefits. A small number of experimental studies suggested modest effects on body weight or metabolic outcomes, while the majority of evidence consisted of case reports and product analyses. Notably, multiple reports described serious adverse health outcomes associated with diet and detox tea consumption, including electrolyte disturbances, cardiovascular events, and liver injury in otherwise healthy individuals. In addition, analytical studies identified undeclared pharmaceutical ingredients, high levels of stimulants, and inconsistencies between labeled and actual product contents in teas marketed as natural. Altogether, current evidence suggests that diet and detox teas marketed for weight loss offer minimal demonstrated benefit and may pose meaningful health risks. The findings highlight important gaps in the evidence base and underscore the need for improved regulatory oversight, clearer labeling, and higher-quality research to better inform consumers and health professionals regarding the use of these products for weight management.

## Graphical abstract

**Figure:**
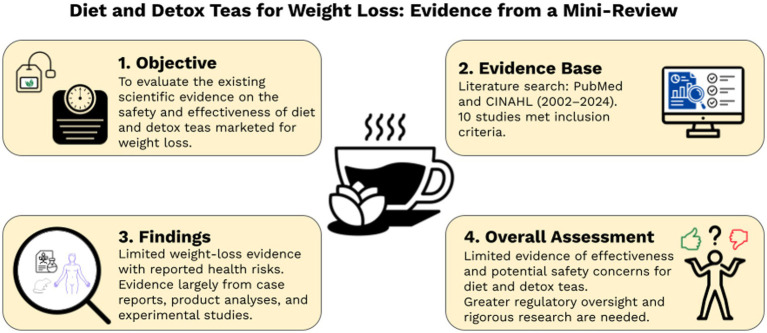

## Introduction

Detoxification, commonly referred to as ‘detox’, is a process of eliminating toxins and cleansing the body to restore physiological balance and promote overall health (1, 2). From ancient practices embedded in religion and culture, such as the use of herbal medicines, meditation, and religious fasting, detoxification has been an integral part of human health traditions. Over time, these practices have evolved into modern approaches emphasizing physical rejuvenation, wellness, and fitness (2).

In recent years, as the global rate of obesity continues to surge, weight loss attempts and efforts among individuals have become equally prevalent (3, 4). The concept of detox diets and supplements has gained popularity, as they claim to cleanse toxins from the body and promote weight loss. Along with the chronic health consequences of being overweight, factors such as social influences and weight-related stigma often drive individuals toward dietary measures that promise rapid results (4, 5). These factors may lead individuals to seek non-prescription alternatives to weight control that are easily available over the counter and perceived as lower risk and relatively lower cost. Among those options, teas marketed as quick and natural remedies for detox and weight loss have emerged as a common choice (1, 6).

Tea has been widely consumed for centuries, valued for its cultural significance and its bioactive compounds, which are associated with various health benefits (7). The bioactive polyphenols present in tea have been associated with health-promoting effects, ranging from potential reductions in chronic disease risk, such as cancer, diabetes, arthritis, and cardiovascular disease, to support for weight management (7, 8). In response to the rising demand for rapid weight-loss supplements, a wide range of teas, marketed as herbal, cleanse, weight-loss, detox, or diet teas, have entered the consumer market (6, 9). These products are widely available in grocery stores and are often promoted with claims to boost metabolism, reduce bloating, flush out toxins, and promote weight loss. Despite being labeled as natural, plant-based, and healthy, the mechanism through which these teas promote weight loss is not clearly supported by scientific evidence, nor are their compositions accurately and consistently disclosed in their labels (1, 6).

In the United States, herbal and dietary supplements are categorized as a special food, which does not subject them to the same strict regulation, safety, and efficacy standards as pharmaceutical medicines, creating regulatory leeway for undeclared ingredients and their quantity to remain unaddressed on product labels (6). Moreover, widespread promotion and endorsement of detox and diet teas through social media, particularly by celebrities and influencers, often includes health claims related to detoxification, rapid weight loss, and “natural” benefits that may not be supported by scientific evidence and may fail to disclose potential risks (2, 6, 10). Marketing practices may therefore contribute to consumer misconceptions regarding the safety and effectiveness of these products. Consistent with this concern, the study by Lai et al. examining online marketing of weight-loss supplements found that many products contained stimulant ingredients and frequently omitted clear safety warnings or regulatory disclaimers (11). Similarly, a content analysis of 12 diet teas sold across three Asian grocery stores in Connecticut found that 91.7% of these teas contained senna leaf, a laxative, while packaging emphasized slimming and weight loss imagery, natural or herbal claims, and regulatory disclaimers that may obscure health risks (12). Such promotional practices may reinforce the perception that these products are safe and effective despite the limited clinical evidence supporting their use.

Although tea consumption offers significant health benefits when consumed in appropriate amounts, the lack of transparent, evidence-based information and messaging in teas marketed for weight loss leaves consumers vulnerable to misinformation and disinformation, posing a broader public health concern. To the best of our knowledge, no comprehensive review has been conducted to assess the safety and efficacy of detox or diet teas marketed for weight loss. Despite several reviews examining detox diets, dietary supplements, or herbal products related to weight management (Table 1), most of them focus on broader detox diet paradigms, general dietary supplements, or herbal products rather than teas marketed specifically for weight loss (1, 2, 7, 10, 13, 14). Consequently, there remains a paucity of synthesized evidence addressing detox and diet teas as a distinct category of products. Given the widespread marketing and consumer use of these teas, a focused synthesis of the available evidence is warranted. To supplement existing knowledge, the current mini-review examines the safety, efficacy, and documented adverse effects associated with detox and diet teas marketed for weight management.

**Table 1:** Selected reviews on detox diets, dietary supplements, and herbal products related to weight management.

Objective of review	Key highlights	Reference
Examine herbal and nutritional supplements used for weight loss	Reported limited and inconsistent evidence supporting herbal supplements for weight management and emphasized the need for stronger clinical studies evaluating safety and efficacy (14).	Nannar et al. (14)
Review supplements used for weight management	Concluded that many commercially available weight-loss supplements lack robust clinical evidence supporting effectiveness and raised concerns regarding safety, labeling accuracy, and regulatory oversight (13).	Mah et al. (13)
Systematically review dietary supplements and alternative therapies used for weight loss	Identified numerous supplements marketed for weight loss but found limited high-quality evidence supporting efficacy and highlighted potential safety concerns associated with their use (10).	Batsis et al. (10)
Examine detox diet paradigms in weight management	Discussed theoretical mechanisms and increasing popularity of detox diets but emphasized that empirical evidence supporting their effectiveness remains limited (2).	Khalil et al. (2)
Evaluate evidence supporting detox diets for toxin elimination and weight management	Found little clinical evidence supporting detox diets for toxin elimination or sustained weight loss and highlighted concerns regarding nutritional adequacy and lack of regulatory oversight (1).	Klein and Kiat (1)
Examine health benefits and potential risks associated with tea consumption	Summarized bioactive compounds in tea and their potential health effects but did not specifically evaluate teas marketed for weight loss or detoxification (7).	Hayat et al. (7)

This review aims to delve into existing literature and evidence to understand the safety and efficacy of these widely marketed yet unregulated detox and diet teas, assessing associated health risks, documented benefits, adverse effects, and identifying the extent to which this subject has been explored by previous research. Given the fragmented nature of the existing evidence, this mini review synthesizes findings across clinical reports, experimental studies, and product analyses to provide a concise assessment of the safety and effectiveness of diet and detox teas marketed for weight loss.

## Methodology

A targeted literature search was conducted in PubMed and CINAHL to identify peer-reviewed studies published in English between January 2002 and December 2024 that examined detox or diet teas marketed for weight loss. These databases were selected because they index the majority of biomedical, clinical, and public health literature related to dietary supplements, nutrition, and consumer health products. Given the exploratory scope of this mini-review and the limited body of literature directly examining detox or diet teas marketed for weight loss, the search strategy was designed to capture peer-reviewed studies most relevant to clinical safety, efficacy, and product composition. Studies were included if they examined the safety, efficacy, composition, or adverse health effects of detox or diet teas marketed for weight management. Studies focusing on unrelated herbal supplements, non-tea weight-loss interventions, or non-English publications were excluded.

**Figure 1:**
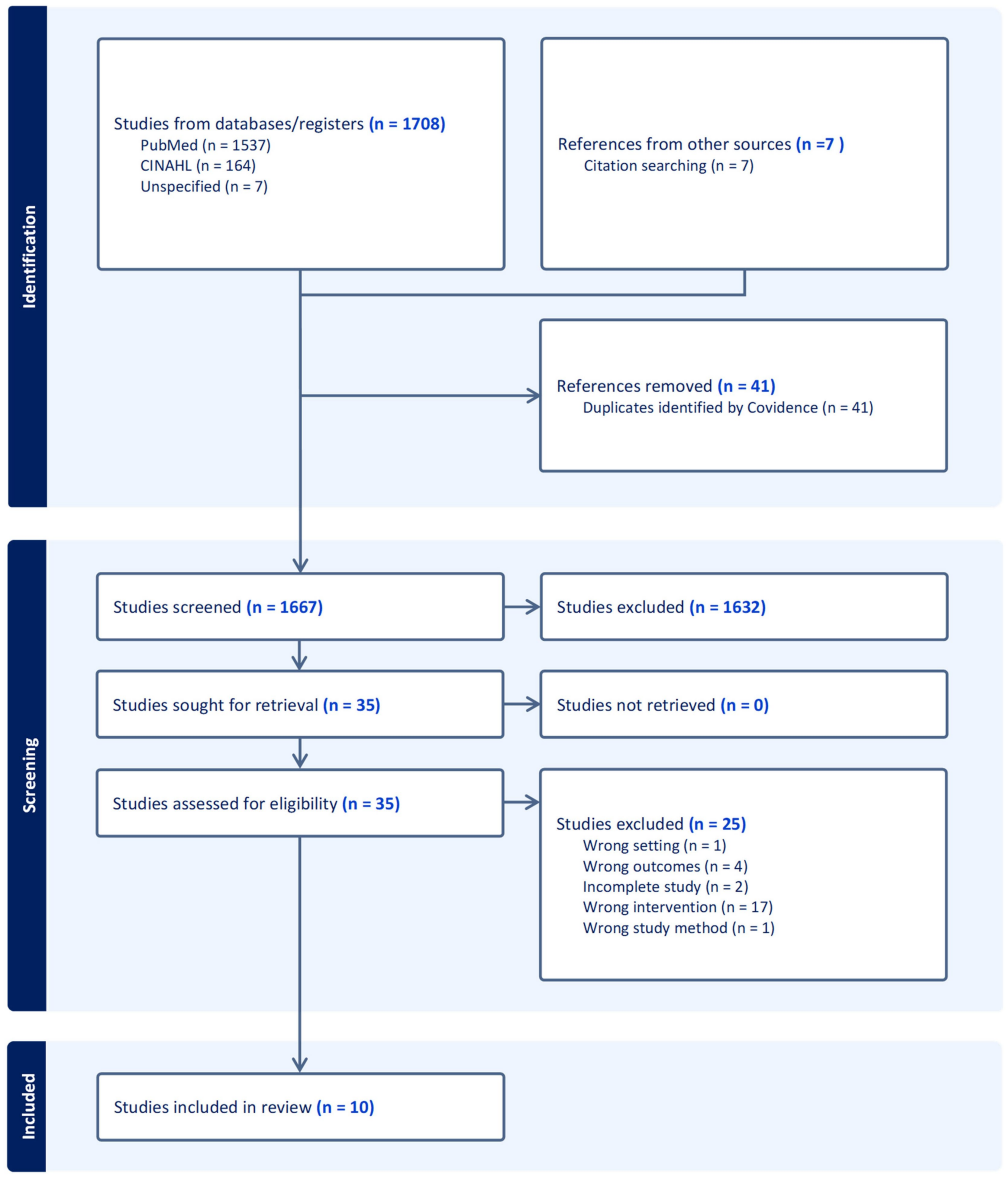
PRISMA flowchart.

All records were imported into Covidence for duplicate removal and screening. Of the 1,708 articles initially retrieved, 1,667 underwent title and abstract screening, followed by full-text review of 35 articles. Ten studies met the inclusion criteria and were included in this review. The screening process is summarized in Figure 1. The relatively small number of eligible studies reflects the limited volume of peer-reviewed research specifically examining detox or diet teas marketed for weight loss.

Data extraction involved compiling key study attributes into a standardized spreadsheet, capturing the author, publication year, study design, population characteristics, intervention type and description, and primary outcomes. The extracted information, summarized in Table 2, was subsequently synthesized to inform the evidence synthesis and address the objectives of this review.

**Table 2:** Summary of extracted study characteristics, interventions, and outcomes.

Study	Author, year	Study design	Population/Sample description	Intervention	Outcomes/results
1.	İsilak et al. (2016) (19)	Case Report	69-year-old overweight patient with controlled hypertension, moderate chronic aortic insufficiency, diastolic murmur, basal rales, and pretibial edema.	Herbal weight loss tea “FORX5” consumed for three weeks for weight loss and appetite suppression.	Developed acute left ventricular dilatation and severe systolic dysfunction with markedly reduced severe left ventricular systolic dysfunction (LVEF) and enlarged LV dimensions despite noncritical coronary lesions; acute LV failure attributed to herbal tea as other common causes were excluded.
2.	Soliman et al. (2018) (18)	Case Report	67-year-old man with no significant past medical history.	Strict five-day kidney cleansing regimen instructing oral intake limited to over 3.7 L fluid daily along with herbal tea.	Presented with weakness, fatigue, nausea, tremor, restlessness and severe hyponatremia; laboratory evaluation revealed serum sodium of 111 mmol/L; diagnosed with acute severe hyponatremia consistent with tea and toast syndrome, required ICU admission and IV isotonic saline, improved after treatment and discontinuation of the regimen.
3.	Gillett et al. (2021) (17)	Case Report	51-year-old previously healthy woman with no regular medications or history of substance use.	Over-the-counter detox tea, approximately two cups daily for four weeks, associated with increasing urinary frequency.	Developed malaise, myalgia, unsteadiness, headache, then an episode of unresponsiveness with vomiting and limb twitching consistent with seizure; found to have severe hyponatremia with neurological symptoms, most likely induced by diuretic effects of the detox tea and associated hypokalemia.
4.	Niazi et al. (2022) (16)	Case Report	36-year-old woman without significant medical, family, or substance-use history.	One month of over-the-counter herbal liver detoxification tea containing burdock root, stinging nettle leaf, cleavers herb, dandelion root, lemon peel, and lemon myrtle leaf.	Presented with one month of cramping abdominal pain and marked cholestatic liver injury with highly elevated ALT, AST, alkaline phosphatase, and bilirubin; liver biopsy showed cholestatic pattern and injury was attributed to Idiosyncratic drug-induced liver injury (DILI) from the tea, with improvement after cessation.
5.	Akçay and Yüksel (2020) (15)	Case Report	41-year-old obese man (BMI 34 kg/m^2^) without coronary risk factors, substance use, or relevant family history.	Consumption of large quantities of mixed teff grain herbal tea (4–5 cups/day for 5 days) marketed for rapid weight loss.	Developed palpitations, confusion, and loss of consciousness followed by ventricular fibrillation and cardiac arrest requiring defibrillation; severe hypokalemia related to teff tea ingestion was identified as the possible cause, with ECG features consistent with arrhythmia from electrolyte disturbance, illustrating that aggressive use of such teas can be attributed to life-threatening cardiac events.
6.	Pascali et al. (2018) (22)	Analytical Laboratory Study	Five commercial slimming herbal supplements samples (teas and soft-gel capsules) marketed as natural plant-based weight loss aids, purchased online and in stores.	Analytical screening of slimming teas and capsules using chromatography/time of flight (Q-TOF LC/MS) and confirmatory liquid chromatography/triple quadrupole (LC–MS/MS) to detect stimulants and anorectic active pharmaceutical ingredients.	Identified caffeine and undeclared sibutramine in two products and other undeclared synthetic drugs; highlighted that herbal weight loss supplements often contain hidden pharmacologically active compounds such as sibutramine, phenolphthalein, bumetanide, and phenytoin that pose significant cardiovascular and neuropsychiatric risks.
7.	Yu et al. (2023) (21)	Animal Experimental Study	Male Sprague–Dawley rats, 8 weeks old, randomized to normal diet, high-fat diet (HFD), or HFD plus Besunyen Slimming Tea (BST) (n = 12 per group).	Oral BST (0.6 g/0.6 kg) given to HFD-fed rats after obesity induction to test anti-obesity and metabolic effects.	Compared with HFD alone, BST reduced waist circumference, food intake, final body weight, body weight gain, BMI, hyperlipidemia, inflammation, insulin resistance, and hepatic lipidosis by enhancing fatty acid oxidation and reducing *de novo* lipogenesis, supporting potential usefulness of BST in obesity and metabolic disorder management (in animal models).
8.	Vidya and Kulkarni (2002) (20)	Uncontrolled Human Intervention Study	35 overweight volunteers aged 30–40 years, 4–6 kg above normal BMI-based weight.	Slim Tea, an herbal tea by Himalaya Drug Company, one 2 g bag taken 2–3 times daily as a hot infusion for 16 weeks, without a specific diet or adjuvant therapy.	All participants lost approximately 1.5–2 kg by two months with improvement in edema and heaviness; tea was well accepted with no reported adverse effects, and authors suggested ingredients such as *Garcinia cambogia* (hydroxycitric acid), *Commiphora mukul*, and *Cyperus scariosus* might contribute to modest weight loss, though findings are limited by small, uncontrolled design.
9.	Lai et al. (2021) (11)	Cross-Sectional Study	51 websites and 105 dietary supplement products marketed as ephedra-containing or ephedra-like substances for weight management.	Evaluation of online supplement labeling for ephedra content, serving size, stimulant ingredients, structure–function weight loss claims, directions, side effects, interaction warnings, diet/exercise advice, and FDA disclaimer.	Findings showed that some products still listed ephedra or ephedrine alkaloids despite the FDA ban, and nearly all contained additional stimulants while frequently omitting clear side-effects, interaction, or FDA disclaimer information; concluded that online ephedra-like weight loss products expose consumers to cardiovascular, CNS, and other adverse risks in an underregulated environment.
10.	Orimadegun et al. (2018) (23)	Quantitative Study	Daily Detox herbal tea from a Nigerian manufacturer, not registered with National Agency for Food Drug Administration and Control (NAFDAC) and with unevaluated clinical claims.	Laboratory quantification of phytochemicals (terpenoids, flavonoids, tannins, phenols, carotenoids, alkaloids, trypsin inhibitor) and micronutrients including trace elements.	Tea contained multiple phytochemicals and micronutrients suggesting potential nutritional value but showed extremely high trypsin inhibitor levels and zinc content exceeding WHO limits for herbal tea, indicating that regular or excessive consumption could antagonize nutrient absorption and adversely affect health.

Given the heterogeneous nature of the included studies, which comprised case reports, laboratory analyses, animal experiments, and a single uncontrolled human intervention study, a formal risk-of-bias tool applicable across all designs was not applied. Instead, study findings were interpreted with consideration of the methodological strengths and limitations inherent to each study design. In particular, case reports were recognized as providing descriptive evidence of potential adverse events but not establishing causation, while uncontrolled intervention and animal studies were interpreted cautiously due to the absence of control groups, limited sample sizes, and challenges in translating experimental findings to human populations.

## Evidence synthesis

### Findings based on study designs

#### Case reports

Multiple case reports describe the harmful health effects of consuming detox and diet teas in individuals. These cases outline the serious risks that are associated with the unregulated use of these teas. For example, a case report documented severe hypokalemia (potassium ~2 mEq/L) and ventricular fibrillation in a 41-year-old man after five days of heavy consumption of a teff grain–based weight-loss tea containing diuretic and laxative herbs such as sage, senna, fennel, and mate (15). In another case, a 36-year-old woman developed marked hepatotoxicity after consuming an over-the-counter liver detox tea. She presented a dramatic elevation in liver enzymes, including an ALT level above 1,300 U/L, and was diagnosed with idiosyncratic drug-induced liver injury. The product contained botanicals such as burdock root, nettle, cleavers, and dandelion root, ingredients not typically associated with hepatotoxicity, suggesting a possible idiosyncratic reaction or undisclosed contaminants (16).

There are also reports of severe hyponatremia associated with detox tea consumption. In one case, a 51-year-old woman developed seizures and an altered mental state after regularly consuming a detox tea containing multiple caffeinated teas and herbal diuretics (17). Her sodium level had fallen to 115 mmol/L. Similarly, a 67-year-old man who consumed more than a gallon of an herbal tea containing uva ursi leaves and juniper presented with a sodium level of 111 mmol/L and required hospitalization (18).

Furthermore, in a case from Turkey, a 69-year-old man developed acute cardiomyopathy after consuming an herbal slimming tea (called “FORX5”). His ejection fraction fell from 65 to 25%, with significant ventricular dilation. Because the tea listed no ingredients, clinicians suspected adulteration with weight-loss drugs associated with cardiotoxicity (19). Taken together, these reports illustrate recurring patterns of electrolyte disturbance, hepatotoxicity, and cardiotoxicity associated with unregulated herbal tea consumption.

#### Experimental studies

Evidence from experimental studies evaluating detox or diet teas is limited and generally characterized by small sample sizes, weak methodology, or lack of replication. One uncontrolled open-label human intervention study identified was an open-label trial of a commercial slimming tea (Slim Tea) involving 35 slightly overweight adults (20). Participants consumed the tea two to three times daily for two months, during which the researchers reported modest weight loss of approximately 1.5–2 kg. No adverse effects were noted, and participants described subjective improvements such as reduced heaviness and absence of edema. The product included ingredients such as Garcinia cambogia, Commiphora mukul, and Cyperus scariosus (20).

Animal research provides additional insight into potential biological activity. In a high-fat diet rat model, Besunyen Slimming Tea (BST) was associated with reduced weight gain and improvements in lipid levels, hepatic fat accumulation, inflammation, insulin sensitivity, and waist circumference (21). These effects were linked to upregulation of fatty acid oxidation pathways (PPAR-*α*, CPT1α) and downregulation of lipogenic regulators (fatty acid synthase, SREBP-1c), suggesting enhanced *β*-oxidation and reduced hepatic lipogenesis through AMPK activation (21). These findings suggest that weight reduction can occur under controlled conditions. This study was funded by the National Natural Science Foundation of China (NSFC), a major public research funding agency, and no commercial sponsors were listed.

Analytical investigations further documented the presence of undeclared substances in teas marketed for slimming or detoxification. High-resolution mass spectrometry identified undeclared synthetic compounds, including sibutramine and caffeine, in products labeled as natural, with prior studies reporting additional adulterants such as phenolphthalein and theophylline (22). Another assessment of online ephedra-like weight-loss supplements found that approximately 11% listed ephedrine alkaloids despite existing regulatory bans. Many products also contained high levels of stimulants, including caffeine (often exceeding 400 mg per recommended daily dose) and yohimbe (11).

#### Findings based on product type: detox vs. diet teas

Detox teas and diet or slimming teas are commonly promoted for different purposes and therefore warrant separate consideration. Detox teas are typically marketed as products intended to cleanse the body or remove toxins and often contain herbal ingredients with diuretic or laxative properties that promote fluid loss. In contrast, diet or slimming teas are generally promoted as products intended to facilitate weight loss through mechanisms such as metabolic stimulation, appetite suppression, or increased fat oxidation, often through stimulant compounds or polyphenol-rich herbal extracts.

#### Detox teas

A study that focused on finding the chemical composition and contamination of a detox tea known as Daily Detox was done in Nigeria (23). This tea was not registered by the National Agency for Food and Drug Administration and Control and has never been assessed in clinical trials. The findings from the study indicate the presence of various phytochemicals, such as phenolics, terpenoids, and flavonoids, and high mineral content, which may contribute to potential health effects. However, it was also found that the tea contained high amounts of trypsin inhibitor and zinc, which can have adverse effects on the body (23). Additional case reports involving detox tea consumption have described adverse outcomes including acute severe hyponatremia and drug-induced liver injury (17, 18, 22, 23).

#### Diet teas

Diet or slimming teas are mostly developed to promote weight loss and to enhance metabolism. Evidence of limited benefits has been found for SlimTea and BST; these were able to produce a measurable decrease in body weight and fat accumulation, due to the activity of polyphenols and flavonoids, which increased fat oxidation (20, 21). Notably, the only human study reporting modest weight loss benefits was conducted by investigators affiliated with the product manufacturer, lacked a control group, randomization, or blinding, and relied largely on subjective outcomes, raising concerns about sponsorship bias and limiting the interpretability and generalizability of its findings (20). In addition, broader regulatory and pharmacologic concerns persist across this product category. Analytical investigations have identified undeclared pharmaceutical agents, including sibutramine, as well as high levels of caffeine in teas marketed as natural weight loss products. Similarly, some supplements continued to list ephedrine alkaloids or related compounds despite existing regulatory bans (11). Such adulterants may provoke sympathomimetic toxicity and have been linked to adverse cardiovascular outcomes (15, 19).

## Discussion

Detox and diet teas are usually marketed as “natural” remedies that promote detoxification and enhance the process of metabolism. However, the current evidence in support of these claims is inconsistent, limited in quantity, and largely derived from study designs that do not permit causal inference. The preclinical work that was done on BST using rat models demonstrated meaningful reductions in obesity markers and hepatic fat accumulation; yet these results cannot be directly applicable to humans, as information on dosing and bioavailability of polyphenols is required (21). Importantly, the sole human study reporting modest weight loss benefits was conducted by investigators affiliated with the product manufacturer and lacked a control group, randomization, or blinding (20). The study also relied largely on subjective outcomes and did not control for diet or physical activity, substantially limiting causal inference and generalizability. Additionally, much of the human evidence available focuses on health risks. There are cases of hepatotoxicity, hyponatremia, and cardiac injury that indicate that using such teas without clinical supervision may cause harm to the liver, kidneys, and electrolyte balance. These negative effects have occurred in healthy individuals, which highlights the fact that the “natural” label can mask the pharmaceutical-like toxicity associated with these teas (15, 17, 18). However, this evidence of adverse reactions comes from single cases and cannot be used to make causal inferences without properly testing these teas in randomized clinical trials. Additionally, from the ephedra marketing and the HRAM-based screening studies, there is information on the presence of undeclared drugs in the teas, but neither of them provided the population-level data required to assess how often consumers are affected (11, 22). In the United States, detox teas are considered dietary supplements, which allows them to be sold in the market without providing any evidence of their safety and efficacy (16).

Another important consideration is the geographic diversity of the products described in the literature. The studies included in this review examined products originating from multiple international contexts, including countries with differing regulatory frameworks for herbal supplements and dietary products. These regulatory environments vary substantially in terms of product formulation, manufacturing oversight, labeling requirements, and market surveillance. As a result, findings from individual case reports or product analyses may not be generalizable to all detox or diet teas available globally or within specific national markets. This heterogeneity highlights the need for stronger regulatory oversight and improved monitoring of products marketed for weight management.

The strength of the available evidence is also constrained by the predominance of lower-level study designs. Most of the included literature consisted of case reports describing adverse health events or analytical studies examining product composition, while only one uncontrolled human intervention study and one animal experiment evaluated potential efficacy outcomes. Although such studies provide valuable preliminary insights, they do not allow causal inference regarding safety or effectiveness. Consequently, the current literature offers limited clinical evidence supporting the weight-loss claims associated with detox and diet teas and highlights the need for well-designed controlled human studies.

Several mechanisms may explain both the perceived benefits and potential harms associated with diet and detox teas. Experimental evidence from the Besunyen Slimming Tea (BST) animal study suggested that certain herbal formulations may influence metabolic pathways related to lipid metabolism, including activation of AMP-activated protein kinase (AMPK) and upregulation of fatty acid oxidation pathways such as PPAR-*α* and CPT1α (19). While these mechanisms are biologically plausible, findings from animal models cannot be assumed to translate directly to meaningful weight-loss effects in humans due to differences in dosing, metabolism, and study conditions. In contrast, many commercially marketed diet or detox teas contain stimulant compounds such as caffeine or ephedra-like substances that may transiently increase metabolic rate and suppress appetite (11, 22). Other products contain laxative or diuretic herbal ingredients such as senna, dandelion, or uva ursi that promote fluid loss rather than sustained reductions in body fat. These mechanisms may produce short-term reductions in body weight that are frequently interpreted by consumers as fat loss. However, the same pharmacologic effects may also contribute to electrolyte disturbances, dehydration, and cardiovascular stress, particularly when consumed in large quantities or in products containing undeclared pharmaceutical agents. Case reports describing severe hyponatremia, hypokalemia, and cardiac complications illustrate the potential risks associated with these products (15, 17, 18).

## Study limitations and future directions

Four limitations were identified in this current literature synthesis. First, the number of available studies specifically examining diet or detox teas marketed for weight loss remains limited. Second, the included studies represent diverse methodologies, including case reports, laboratory analyses, animal experiments, and small experimental studies, which limit direct comparison across findings and restrict the ability to draw causal conclusions regarding safety or effectiveness. Third, substantial heterogeneity exists in the composition and formulation of commercially available detox and diet teas, meaning findings from individual studies may not be generalizable across products or geographic markets. Finally, the literature search was conducted using two databases, PubMed and CINAHL, which were selected because they index a large proportion of biomedical and public health literature. However, relevant studies indexed in other databases may not have been captured, and therefore the possibility of additional studies cannot be entirely excluded.

Future research should focus on conducting independently funded and transparent studies that evaluate the composition, benefits, and adverse effects of the commonly used detox and diet teas. It is of utmost importance that regulatory agencies monitor the online marketing activities associated with these teas and that routine analytical testing of these weight loss products is ensured. Moreover, there is a necessity for qualitative studies that assess the reasons why consumers continue to view detox teas as harmless despite evidence of risks. To maintain scientific integrity, all research done in this area should disclose its funding sources and conflicts of interest.

## Conclusion

The evidence synthesized in this review shows that detox and diet teas marketed for weight loss do not have sufficient evidence to be regarded as safe and effective, despite being marketed for this purpose. Evidence of their benefits in humans is extremely limited, with only one Slim Tea trial demonstrating slight weight loss, and an animal intervention showing metabolic benefits that do not provide adequate evidence of effectiveness in humans. In contrast, multiple case reports link these products to serious adverse health effects, including hepatotoxicity, severe hyponatremia, hypokalemia, cardiac arrhythmias, and acute cardiac dysfunction, often in individuals without significant underlying health conditions. Analytical studies further highlight the presence of undeclared drugs such as sibutramine, phenolphthalein, bumetanide, phenytoin, and stimulants such as ephedrine or ephedrine-like compounds, caffeine, and excessive levels of trace elements in teas promoted as natural or herbal, along with incomplete or misleading labeling. With regulatory frameworks treating these products as dietary supplements and not medicines, manufacturers are not required to demonstrate their safety, efficacy, or consistent quality before marketing. At the same time, promotional messaging for these products continues to emphasize detoxification, rapid weight loss, and natural benefits that are not clinically evident. The findings of this review raise concerns about product quality, effectiveness, and potential health risks and highlight the need for stronger regulatory oversight, transparent and accurate labeling, and proper evidence before these teas can be responsibly recommended for weight management. Greater regulatory scrutiny, clearer labeling requirements, and independent research are necessary to ensure that consumers and healthcare professionals have reliable information about the safety and effectiveness of these products. Until more rigorous clinical evidence and stronger regulatory oversight are available, current evidence is insufficient to support the safety or effectiveness of diet and detox teas as strategies for weight management.
